# Intergenomic and epistatic interactions control free radical mediated pancreatic β-cell damage

**DOI:** 10.3389/fgene.2022.994501

**Published:** 2022-10-07

**Authors:** Jing Chen, Renhua Li, Sarah Knapp, Guizhi Zhu, Robert L. Whitener, Edward H. Leiter, Clayton E. Mathews

**Affiliations:** ^1^ Department of Pathology, Immunology, and Laboratory Medicine, University of Florida, Gainesville, FL, United States; ^2^ Henry M Jackson Foundation for the Advancement of Military Medicine (HJF), Bethesda, MD, United States; ^3^ The Jackson Laboratory, Bar Harbor, ME, United States

**Keywords:** diabetes, free radical, mouse model, epistasis, mitochondrial genome, beta cell (β cell), alloxan

## Abstract

Alloxan (AL)-generated Reactive Oxygen Species (ROS) selectively destroy insulin-producing pancreatic β-cells. A previous genome-wide scan (GWS) using a cohort of 296 F2 hybrids between NOD (AL-sensitive) and ALR (AL-resistant) mice identified linkages contributing to β-cell susceptibility or resistance to AL-induced diabetes on Chromosomes (Chr) 2, 3, 8, and a single nucleotide polymorphism in *mt-Nd2* of the mitochondrial genome (mtDNA). AL treatment of congenic and consomic NOD mouse stocks confirmed resistance linked to both the mtDNA and the Chr 8 locus from ALR [NOD.mt^ALR^.ALR-(*D8Mit293-D8Mit137*)]. To identify possible epistatic interactions, the GWS analysis was expanded to 678 F2 mice. ALR-derived diabetes-resistance linkages on Chr 8 as well as the *mt-Nd2* ^*a*^ allele were confirmed and novel additional linkages on Chr 4, 5, 6, 7, and 13 were identified. Epistasis was observed between the linkages on Chr 8 and 2 and Chr 8 and 6. Furthermore, the *mt-Nd2* genotype affected the epistatic interactions between Chr 8 and 2. These results demonstrate that a combination of nuclear-cytoplasmic genome interactions regulates β-cell sensitivity to ROS-mediated ALD.

## Highlights

• Reactive oxygen species play important role in the beta cell death that accompanies in both Type 1 and Type 2 diabetes
• We utilized an alloxan-induced diabetes mouse model to determine the genetic contributions to beta cell death
• Beta cell sensitivity to free radicals is controlled by both the nuclear genome and a polymorphism in the mitochondrial gene, *mt-Nd2*
• Epistatic and intergenomic interactions regulate beta cell resistance to free radicals

## Introduction

Alloxan (AL) is a potent free radical/reactive oxygen species (ROS) generator and selective pancreatic β-cell toxin in mice and rats. In these species β-cells are especially sensitive to ROS because of their low levels of antioxidative capabilities (Lenzen et al., 1996; Tiedge et al., 1997; Mathews et al., 2001); however, the genetic determinants of β-cell sensitivity to ROS are not well understood. Young prediabetic NOD/ShiLtJ (NOD) mice are sensitive to a threshold AL dose whereas mice of the closely related ALR/LtJ (ALR) strain, selected for AL resistance, maintain strong resistance (Ino et al., 1991). Indeed, pancreatic islet cells of the ALR strain, unlike those of autoimmune diabetes-prone NOD strain, are remarkable in their resistance to a broad variety of diabetogenic stresses, including ROS, proinflammatory cytokines, and immune cell-mediated destruction (Ino et al., 1991; Mathews et al., 2001; Mathews et al., 2003). The differential strain sensitivity to AL-mediated diabetes (ALD) has previously been employed to probe the underlying genetic control of β-cell susceptibility/resistance to ROS-mediated damage. A preliminary analysis using an F2 cohort of 296 mice linked β-cell ALD resistance to loci in both the nuclear and mitochondrial (mtDNA) genomes (Chen et al., 2008b). Nuclear loci contributing to susceptibility or resistance were identified on Chromosomes (Chr) 2, 3, and 8.

Mitochondria (mt) are important sources of ROS generation (Mathews et al., 2005; Gusdon et al., 2007; Chen et al., 2008b; Gusdon et al., 2008). The mt electron transport chain proteins are encoded by genes in both nuclear and mt genomes therefore, normal mt function requires the activity of both genomes. Although mtDNA mutations and/or polymorphisms have been attributed to many human diseases including diabetes (Mathews et al., 1999; Mathews et al., 2005; Wallace, 2010), the interaction between mtDNA and nuclear genome in the pathogenesis of diabetes has not been extensively studied. The only polymorphism distinguishing the mtDNA of ALR from NOD is a single nucleotide polymorphism (SNP) at mtDNA position 4,738 (C4738A) in *mt-Nd2*, NADH dehydrogenase subunit 2, resulting in a leucine to methionine amino acid substitution at residue 276 (L276M) in ALR (Mathews et al., 2005). The NOD allele is denoted as *mt-Nd2* ^*c*^ and the ALR allele as *mt-Nd2* ^*a*^ (Mathews et al., 2005). This unique difference in the mtDNA allows the analysis of mt-nuclear interactions in a manner linking nuclear loci to *mt-Nd2*. To define additional linkages as well as explore epistasis and intergenomic nuclear-mt interactions, we expanded the GWS study cohort to 678 F2 mice. Here we demonstrate that ALD susceptibility is a polygenic trait entailing epistatic interaction between the nuclear and mt genomes.

## Methods

### Mice

NOD/ShiLtJ, NOD.129S7(B6)-*Rag1* ^*tm1Mom*^ */J* (NOD.*Rag1* ^*−/−*^), and ALR/LtJ (ALR) mice were bred and maintained in our specific pathogen-free animal facility at the University of Florida. Progeny of reciprocal F1 matings between the NOD and ALR strains were intercrossed in 4 parental combinations, as described previously (Chen et al., 2008b). In the current study, the total number of F2 individuals used for the genome-wide scan (GWS) was 678, including the 296 previously reported (Chen et al., 2008b). NOD. ALRc8 congenic mice, with a long congenic segment from *D8Mit293* (36.2 Mb) to *D8Mit137* (103.9 Mb) were created as previously described (Chen et al., 2008a). Interval specific Chr 8 congenic mice were generated by outcrossing NOD. ALRc8 (*D8Mit293*-*D8Mit137*) mice to the parental NOD strain, typing the progeny with microsatellite markers throughout this interval, and selecting recombinants for ALR markers between *D8Mit293* and *D8Mit137*. This resulted in the establishment of two sub-congenic lines: NOD. ALRc8 (*D8Mit293-D8Mit74*) and NOD. ALRc8 (*D8Mit268*-*D8Mit137*). These shortened congenic regions flank 36.2 Mb–77.8 Mb and 88.3 Mb–103.9 Mb on Chr 8, respectively. All procedures described within this manuscript were approved by the University of Florida’s Institutional Animal Care and Use Committee (UF-IACUC 201905476) and carried out in accordance with the National Institutes of Health guide for the care and use of Laboratory animals.

### Alloxan injection and diagnosis of diabetes

Freshly prepared alloxan monohydrate (Sigma, St. Loius, MO) was administered i.v. to both male and female F2 mice as well as male congenic mice through the tail vein at 6 weeks of age (Chen et al., 2008b). All F2 mice in the GWS received the dose of 52 mg/kg. Congenic mice were subjected to a dose response where AL was administered at the escalating doses of 45 mg/kg, 50 mg/kg, or 55 mg/kg. Blood from non-fasted mice was drawn immediately prior to the injection of AL, and then at 7 and 14 days post-AL administration. Glucose levels were measured with a One Touch Ultra 2 glucometer (Life Scan, Milpitas, CA). Alloxan-induced diabetes (ALD) was diagnosed in mice with blood glucose of ≥ 250 mg/dl (Chen et al., 2008b).

### DNA extraction and genotyping

DNA was extracted from kidney and a GWS performed using a total of 80 microsatellite and 81 single nucleotide polymorphism (SNP) markers, as described previously (Chen et al., 2008b). Physical positions of markers were based on Mouse Genome Informatics at The Jackson Laboratory (http://www.informatics.jax.org/) and NCBI build 37 of the mouse genome (http://www.ncbi.nlm.nih.gov/projects/genome/guide/mouse/).

### Quantitative trait loci and statistical analyses

Quantitative Trait Loci (QTL) linkage analysis was performed using R/qtl (The Jackson Laboratory, Bar Harbor, ME. http://www.rqtl.org/) (Broman et al., 2003). Thresholds for LOD scores are based on 1,000 permutation tests (Churchill and Doerge, 1994). In this intercross the values of LOD score thresholds were 2.23 and 3.27 for suggestive (*p* < 0.63) and significant (*p* < 0.05) levels, respectively, when sex was included as an additive covariate. Confidence intervals of QTLs were determined by using the posterior probability density method (Chen and Kendziorski, 2007). Chi-squares and *p* values were calculated using JMP software (SAS Institute Inc.).

### Transcriptome analysis

Transcriptome analysis was performed after isolation of RNA from isolated islets using Affymetrix Genechip Mouse Gene 2.0 ST array. Pancreatic islets from 10–12-week-old NOD.ALRc8 (*D8Mit293*-*D8Mit137*) and NOD.*Rag1* ^*−/−*^ mice were isolated as previously described (Whitener et al., 2017). As a broad range of insulitis severity is seen at this age range in NOD mice, immunodeficient NOD-*Rag1* ^−/−^ mice that do not develop insulitis or T1D were used *in lieu* of NOD mice. Pancreata from two mice were used to isolate pools of 200 and 250 islets per strain. At least three pools of islets were generated for each mouse strain. RNA was extracted for islets using the Qiagen RNeasy kit according to the manufacturer’s instructions (Qiagen, Hilden, Germany). All extractions were performed inside a benchtop hood to minimize the potential for RNAse contamination and included on-column DNA digestion. Quality of the extracted RNA was verified using the Bio-Rad Experion StdSense RNA kit (Bio-Rad, Hercules, CA). All of the samples had RQI values above 6.5. As we used whole tissue comprised of multiple cell types, significance thresholds for differential expression were initially conservatively set at |log2(FC)|> 0.5 with a *p*-value of ≤ 0.05.

## Results

### The maternally-derived ALR mtDNA Nd2^a^ allele contributes to ALD resistance

Our previous results have demonstrated that ALR females and males resist ALD while NOD females and males are susceptible to ALD development at a dose of 52 mg/kg (Mathews et al., 2005; Chen et al., 2008b). At this dose, F1 hybrid progeny of both sexes from crosses of ALR females to NOD males [(RxD)F1] resist ALD while their counterpart (DxR)F1 mice do not. As previously reported, spontaneous diabetes did not occur in any of the reciprocal F1 hybrids (Mathews et al., 2005; Chen et al., 2008b). The incidence of ALD was 23.2% (157 out of 678) in the total cohort of reciprocal F2 mice (Table 1: both sexes and all cross directions combined) and remaining close to our reported results using a smaller F2 population [62 out of 296 (20.9%) (Chen et al., 2008b)]. Males exhibited a two-fold higher incidence of ALD compared to females (30.9% versus 15.4%). Stratification of the results by cross direction and sex demonstrated that F2 male mice with both the mtDNA and Chr Y derived from NOD had the highest ALD incidence (47.8%) whereas males with the mtDNA and Chr Y from ALR associated with the lowest ALD incidence (17.5%). The SNP at mtDNA position 4,738 in *mt-Nd2*, the only sequence difference in the mtDNA of ALR versus NOD (Mathews et al., 2005), remained a significant linkage where the ALR-derived allele, *mt-Nd2* ^*a*^, provided protection against ALD (Chi-square of 21.2; *p* < 0.0001). All F2 mice with *mt-Nd2* ^*c*^ had twice the risk of developing ALD [30.4% incidence (107 out of 353)] compared to a 15.4% (50 out of 325) incidence in mice inheriting *mt-Nd2* ^*a*^. This confirmed our previous findings (Mathews et al., 2005) that the SNP variation resulting in a leucine to methionine amino acid substitution in ND2 is an important mediator of β-cell sensitivity to ALD (Chen et al., 2008b).

**TABLE 1:** Incidence of alloxan-induced diabetes in each direction of the F2 cohort.

F1 intercross	Total incidence	Female incidence	Male incidence	Y Chr	mtDNA
DRXDR	21.5% (39/181)	12.9% (12/93)	30.7% (27/88)	R	D
RDXRD	16.4% (27/165)	8.2% (7/85)	25.0% (20/80)	D	R
DRXRD	39.5% (68/172)	30.0% (24/80)	47.8% (44/92)	D	D
RDXDR	14.4% (23/160)	11.3% (9/80)	17.5% (14/80)	R	R

#### ALD is a polygenic phenotype under control of multiple nuclear loci

**FIGURE 1:**
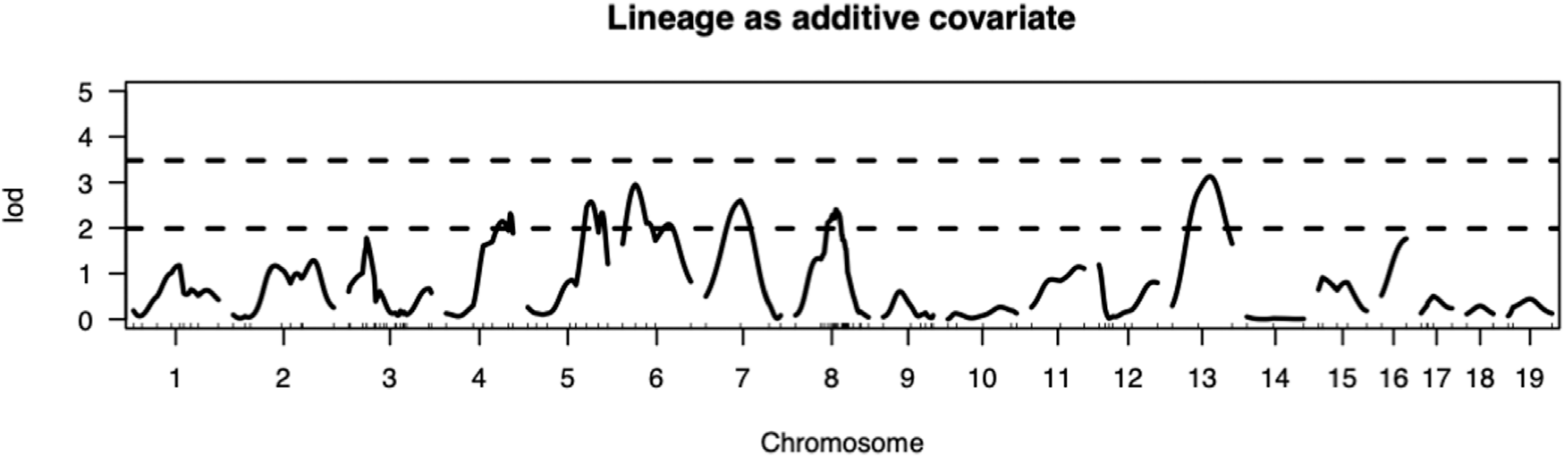
Genome-wide analysis revealed QTLs on Chromosomes 4, 5, 6, 7, 8, and 13. Thresholds for LOD scores are based on 1,000 permutation tests. In this intercross the values of LOD score thresholds are 2.23 and 3.27 for suggestive (*p* < 0.63) and significant (*p* < 0.05) levels, respectively, when sex is included as an additive covariate and mitochondrial lineage added as additive covariate.

**FIGURE 2:**
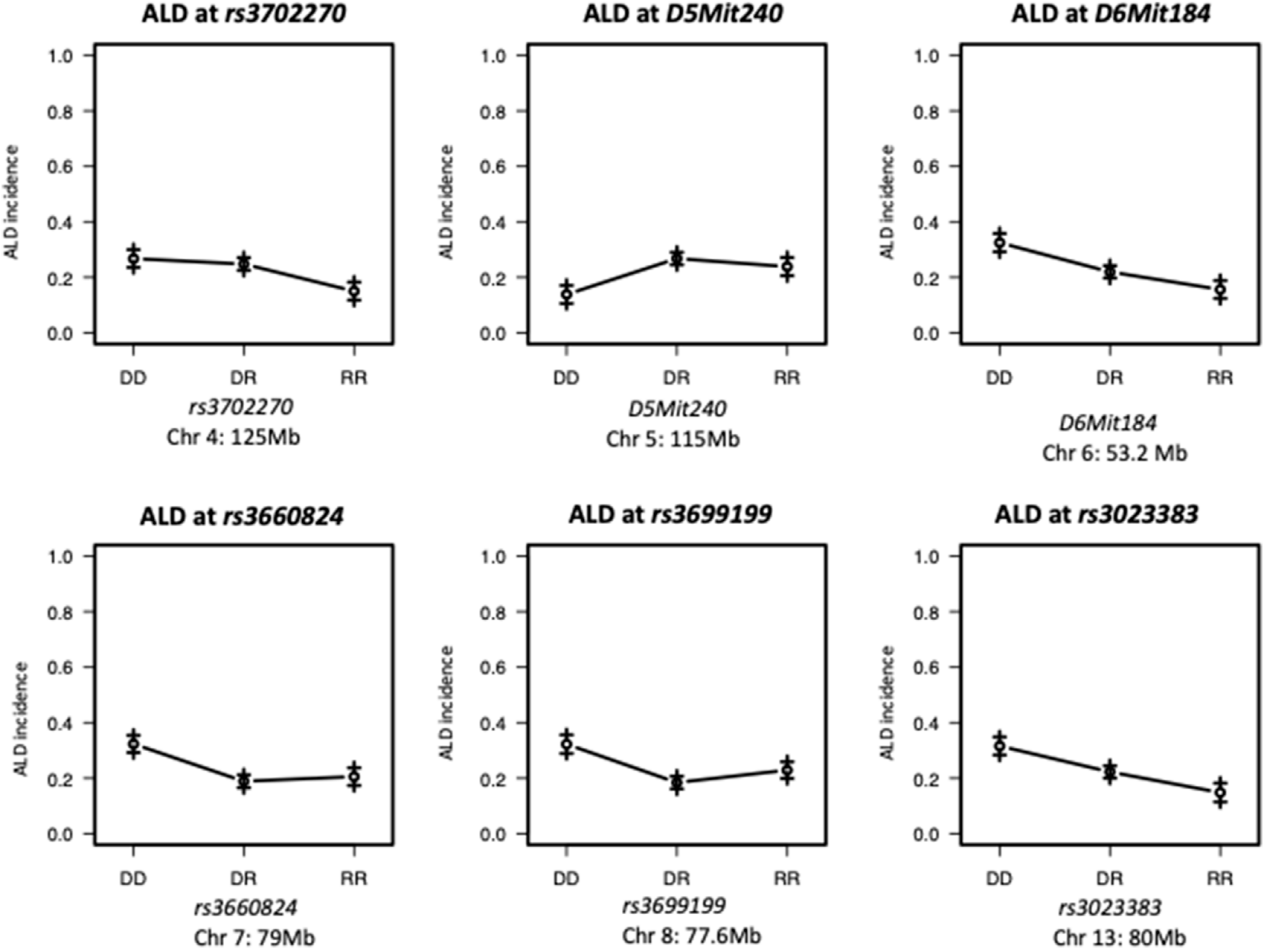
Effect Plots of markers on the peak of each chromosome. ALR allele on Chromosome 4 and 7 recessively contributes to resistance; ALR allele on Chromosomes 8 dominantly contributes to resistance. ALR allele on Chromosome 5 dominantly contributes to susceptibility; On Chromosomes 6 and 13, the susceptibility contributed by NOD allele and resistance contributed by ALR allele co-dominantly exist. DD: homozygous for NOD; DR: heterozygous for NOD and ALR; RR: homozygous for ALR.

The present study utilizing a larger segregating population provided additional power to confirm previous contributions to ALD from the nuclear genome and to detect additional linkages (Figures 1,2; Table 2). In this larger cohort, GWS revealed suggestive linkages (with a LOD score threshold of ≥ 2.2) where ALR genome contributed resistance on Chr 4 (dominant, χ^2^ = 10.9, *p* = 0.0123), Chr 6 (co-dominant, χ^2^ = 12.7, *p* = 0.0017), Chr 7 (dominant, χ^2^ = 11.9, *p* = 0.0078), Chr 8 (dominant, χ^2^ = 12.1, *p* = 0.0023), and Chr 13 (co-dominant, χ^2^ = 12.4, *p* = 0.0021). Interestingly, the ALD-susceptible NOD genome provided a resistance locus on Chr 5 (recessive, χ^2^ = 11.9, *p* = 0.0026). Among these 5 loci, only the linkage on Chr 8 was previously reported as associated with ALD using a smaller cohort (Chen et al., 2008b). GWS with the larger cohort failed to confirm a locus on Chr 3 where a susceptibility allele was unexpectedly contributed by ALR (Chen et al., 2008b). Additionally, whereas *rs3681744* on Chr 2 had contributed negative heterosis in our previous study (Chen et al., 2008b), it was not confirmed in the larger cohort as an independent linkage. However, this region was identified (as described below) as an interacting locus with Chr. 8 and *mt-Nd2*.

**TABLE 2:** Loci that are significantly linked to ALD resistance or susceptibility.

Chromosome	Maker	Position (Mb)	Chi square	*p*	Effect plot^†^
mt	*mt-Nd2*		21.2	< 0.0001	R-resistance
4	*rs3702270*	121	10.9	0.0123	R/R-resistance
5	*D5Mit240*	109.5	11.9	0.0026	R/D-susceptibility
6	*D6Mit184*	53.2	12.7	0.0017	R/R-resistance
					R/D-intermediate
7	*rs3660824*	69	11.9	0.0078	R/R and R/D-resistance
8	*rs3699199*	82.3	12.1	0.0023	R/R and R/D-resistance
13	*rs3023383*	61.1	12.36	0.0021	R/R-resistance
					R/D-Intermediated

^†^For effect plot R indicates the ALR, allele and D indicates the NOD, allele.

### Sub-congenic analysis reduced the region of Chromosome 8 providing resistance to ALD

**FIGURE 3:**
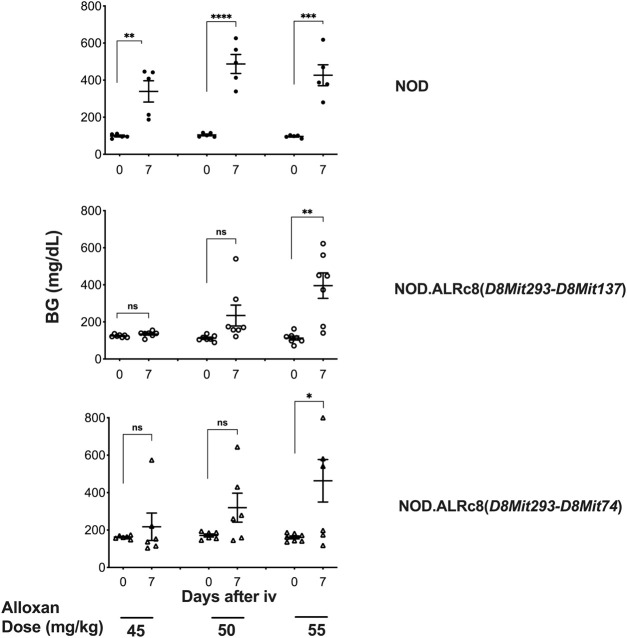
Mice congenic for ALR on chromosome 8 with short and long congenic regions show the same resistance to alloxan. Six-week-old male mice were challenged with different doses of alloxan as indicated. Blood glucose were measured before and 7 days after alloxan treatment. Upper panel: NOD control; middle panel: NOD. ALRc8 (*D8Mit293-D8Mit137*); lower panel: NOD. ALRc8 (*D8Mit293-D8Mit74*). *n* = 5–7. Unpaired *t* test. *p < 0.05; **p < 0.01; ***p < 0.001; ****p < 0.0001.

**FIGURE 4:**
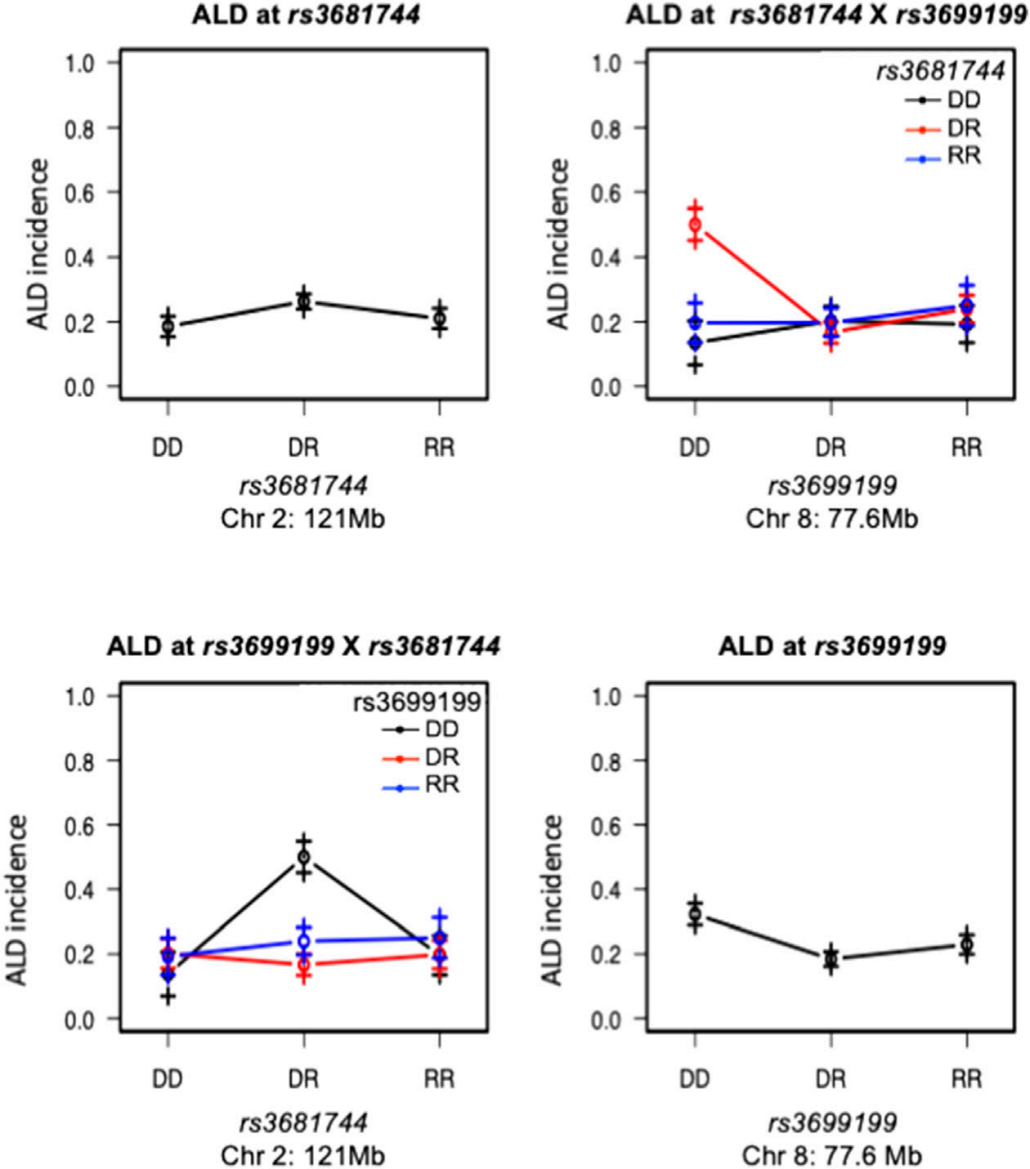
Interaction between Chromosomes 2 and 8 was plotted for ALD incidence. Tope left and lower right panels are effect plot of Chr 2 and Chr 8 respectively, same as in Figure 2. Top right panel: Chr 8 genotype is on the *x*-axis, Chr 2 genotype is shown as group points. ALR allele on Chr 8 dominantly suppresses the sensitivity, therefore the highest ALD incidence is seen in the co-existence of homozygous NOD genotype on Chr 8 and heterozygosity on Chromosome 2; Bottom left panel, showing the same interaction result with Chr 2 genotype on the *x*-axis and Chr 8 genotype as group points. The interaction is significant as shown in Table 3. DD: homozygous for NOD; DR: heterozygous for NOD/ALR; RR: homozygous for ALR.

**FIGURE 5:**
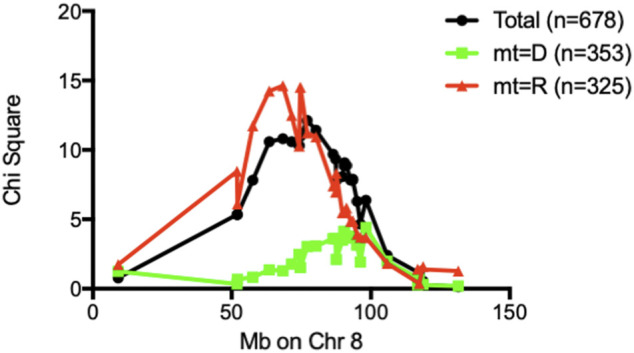
Chi-square values of each marker on Chr 8 were plotted against marker position in Mb. When stratified by mt-genome, the linkage existed only when mt-genome is ALR derived *mt-Nd2* ^a^. Black line: Whole population of F2 (*n* = 678); Red line: population of F2 mice with ALR-derived *mt-Nd2* ^*a*^ (*n* = 325); Green line: population of F2 mice with NOD-derived *mt-Nd2* ^*c*^ (*n* = 353).

We have previously reported that a Chr 8 congenic line, designated NOD. ALRc8 (*D8Mit205-D8Mit137*) and generated by introgressing ALR Chr 8 genetic markers into the NOD genome between 50.8 Mb and 103.8 Mb, demonstrated resistance to ALD (Chen et al., 2008b). Further backcrossing to NOD of this long congenic line allowed generation of a shorter sub-congenic strain, NOD. ALRc8 (*D8Mit293*-*D8Mit74*) (36.2 Mb–77.8 Mb) that partially overlaps with the long congenic line to reduce the size of this ALR-derived protective genetic region. When challenged with different doses of AL, this short congenic line demonstrated the same resistance as the long congenic line (Figure 3). Thus, the protective locus is located in the proximal region between 50.8 Mb and 77.8 Mb. Consistently, the peak for linkage on Chr 8 is in this region at 77 Mb (Figures 1, 4, 5).

### Epistatic interactions contribute to sensitivity of pancreatic β-cells to free radical induced damage

Interaction between unlinked loci was tested using R/qtl, fitting multiple regression models and dropping one QTL at a time. In addition to confirmation of linkages to *mt-Nd2* (C4738A genotyping), Chr 4, 5, 6, 7, 8, and 13 (Figure 1) that were detected in the single QTL scan, a significant interaction between Chr 2 and 8 was detected (Table 3; Figure 4). The highest ALD incidence was observed in mice with the combination of DD genotype at marker *rs3699199* on Chr 8 and a heterozygous genotype (DR) at marker *rs3681744* on Chr 2 (Figure 4). While this Chr 2 linkage was not detected as a single QTL as it did not reach significance (Figure 1), the current GWS does confirm that *rs3681744* contributes overdominance to ALD but only when interacting with homozygous NOD genome marked by *rs3699199* on Chr 8.

**TABLE 3:** Multiple regression model, drop one QTL at a time ANOVA table.

	df	Type III SS	LOD	%var	F value	*p*-value (Chi^2^)	*P*-value(F)	
sex	1	2.903	4.354	2.428	19.719	7.54E-06	1.05E-05	***
mt-genome	1	2.076	3.127	1.737	14.105	1.48E-04	0.000188	***
4@125.4	2	1.995	3.006	1.668	6.775	0.001	0.001223	**
5@114.7	2	1.354	2.047	1.133	4.599	0.009	0.010384	*
6@53.2	2	1.72	2.595	1.438	5.841	0.003	0.003058	**
7@78.7	2	2.683	4.028	2.244	9.112	9.37E-05	0.000125	***
8@77.2	6	5.31	7.869	4.441	6.012	2.48E-06	3.90E-06	***
13@80.3	2	1.772	2.673	1.482	6.019	0.002	0.002569	**
2@121.4	6	4.522	6.727	3.782	5.119	2.56E-05	3.74E-05	***
8@77.2:2@121.4	4	3.511	5.25	2.937	5.963	7.36E-05	0.000102	***

***p < 0.001; **p < 0.01; *p < 0.05. Numbers after at denote marker position on the chromosome (numbers before at) in Mb.

### Intergenomic interactions affect sensitivity to ALD

**FIGURE 6:**
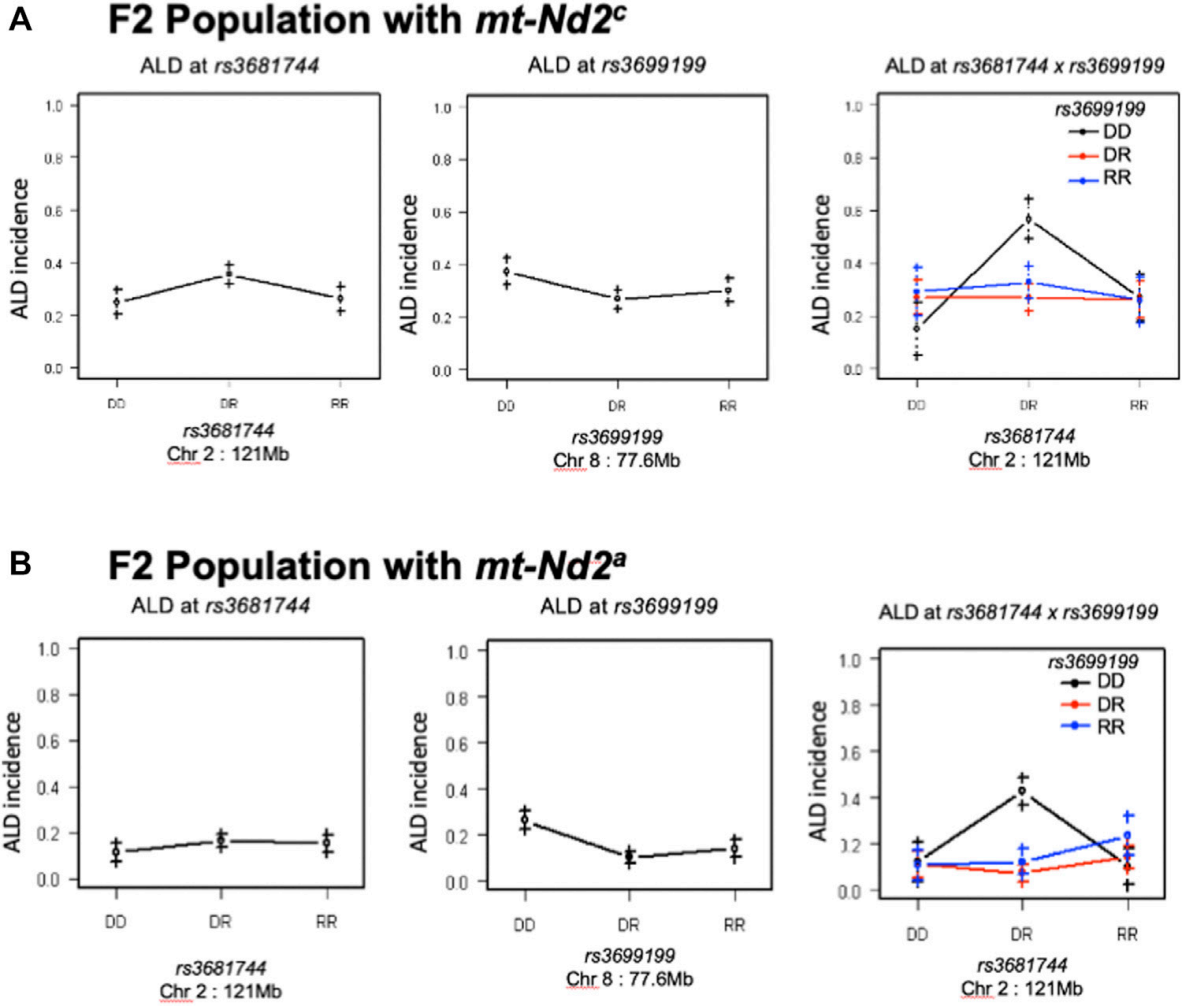
The interaction between Chr 8 and 2 were analyzed in 2 subgroups according to mt-genotype. Panel **(A)**, interaction in the subgroup of mice with *mt-* *Nd2* ^*c*^ type. Left and middle are the effects of QTL on Chr 2 and 8 respectively. Right plot shows their joint effects with chr 2 genotypes on the *x*-axis and chr 8 genotypes indicated by grouping of points (DD, black; DR, red; and RR, blue). Panel **(B)**, the same analysis as in Panel A was done in the group of mice with *mt-* *Nd2* ^*a*^ type.

An analysis was performed to calculate Chi-squares for each locus tested based on mt genotype. This investigation identified an interaction between *mt-Nd2* ^*a*^ and *rs3699199* on Chr 8 [Figure 2 (effect plot) and Table 2] that provides dominant ALD resistance. In fact, when stratified by mt-genome, the linkage on Chr 8 exists only when mt-genome is ALR derived *mt-Nd2* ^a^ (Figure 5). Further, the intragenomic epistasis between QTL on Chr 8 and 2 was affected by the *mt-Nd2* ^*c*^ genotype (Figure 6). Here it was identified that mice with *mt-Nd2* ^*c*^ exhibited increased ALD susceptibility with an overall incidence of 57% when heterozygous for *rs3681744* on Chr 2 in combination with homozygosity for the D genotype at *rs3699199* on Chr 8 (Figure 6A). In contrast, ALR alleles on Chr 8 dominantly contribute to resistance in F2 mice with the ALR mtDNA, thus reducing ALD incidence in all genotype combinations, including when *rs3681744* is heterozygous and *rs3699199* is DD, where incidence decreased to 40% (Figure 6B). The different degree of the Chr 8-2 interaction in the two cohorts with different allotypes of *mt-Nd2* implicates the important contribution of intergenomic interactions to ALD susceptibility.

**FIGURE 7:**
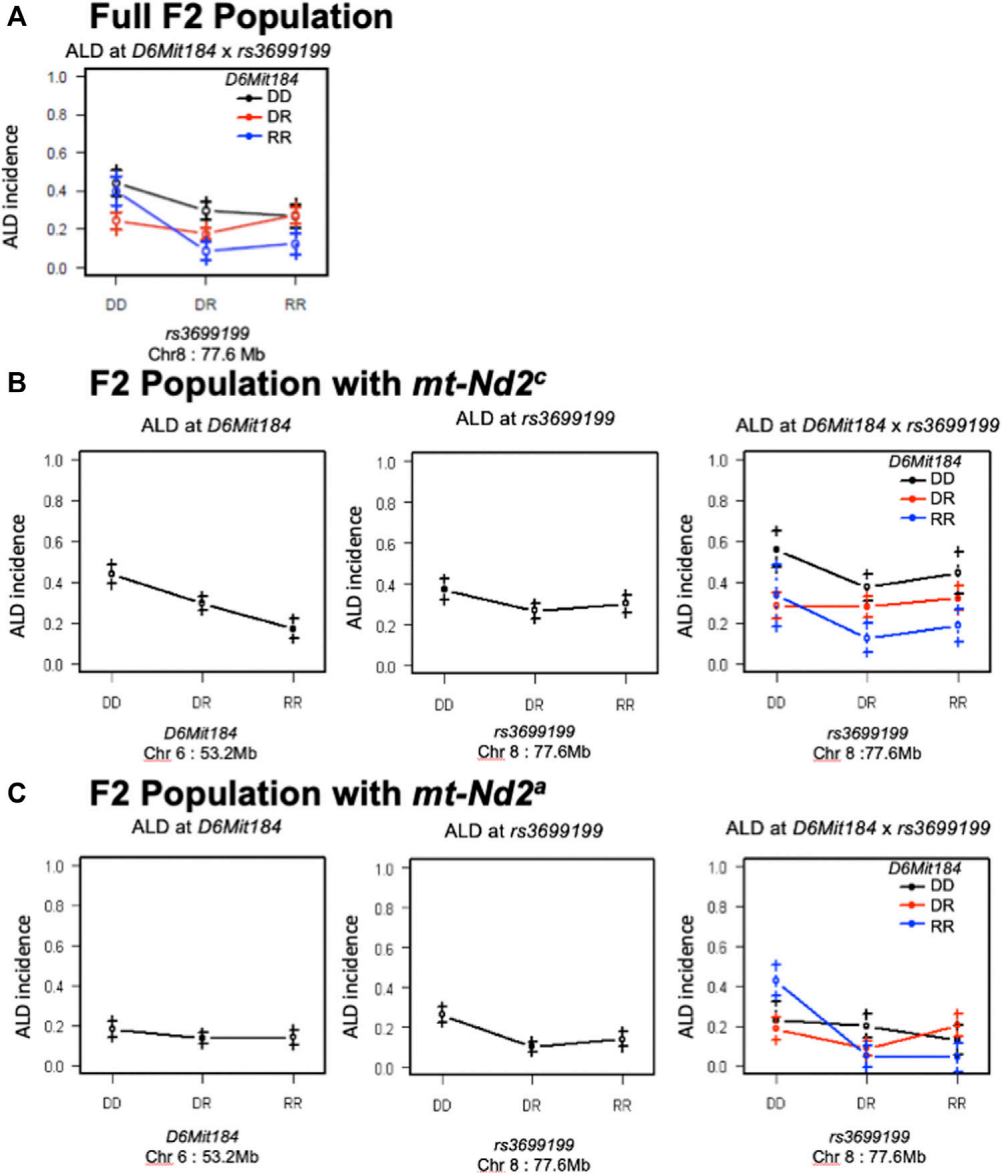
The interaction between Chr 8 and 6 were analyzed in 2 subgroups according to mt-genotype. Panel **(A)** shows the interactions of Chr 8 and Chr 6 in the full population of F2 mice. Panel **(B)**, interaction in the subgroup of mice with NOD derived *mt-Nd2* ^*c*^ type. In this subgroup, coexistence of homozygous DD type on both chromosomes denotes the highest susceptibility to ALD; panel **(C)**, interaction in the subgroup of mice with *mt-Nd2* ^*a*^ type. In this group, homozygous DD on Chr 8 and RR on Chr 6 confer the highest susceptibility to ALD.

Likewise, the interaction between QTLs on Chrs 8 and 6 was highly dependent on the genotype at mtDNA nt 4,738 (Figure 7). Figure 7A elaborates the interactions of Chr 8 and Chr 6 in the full population of F2 mice (both mt populations present). Here the absence of ALR genome on Chr 8 allows the penetrance of the ALD susceptibility phenotype regardless of Chr 6 genotype. In the group of mice with *mt-Nd2* ^*c*^ allele (Figure 7B) the co-existence of DD on both Chromosomes 6 and 8 contributes to the highest incidence of ALD. Stratification for the ALR-derived *mt-Nd2* ^*a*^ allele, in comparison to the NOD-derived *mt-Nd2* ^*c*^ allele, showed suppressed action of homozygous NOD alleles on Chr 6 and Chr 8 respectively (Figure 7B,C). However, in the ALR-derived *mt-Nd2* ^*a*^ group, combination of NOD homozygosity (DD) on Chr 8 and ALR homozygosity (RR) on Chr 6 (Figure 7C) unexpectedly showed enhanced sensitivity.

### Genes differentially expressed in islets from insulitis-free NOD-Rag1−/− and NOD.ALRc8(*D8Mit293-D8Mit137*) mice may confer inter-genomic interaction

**FIGURE 8:**
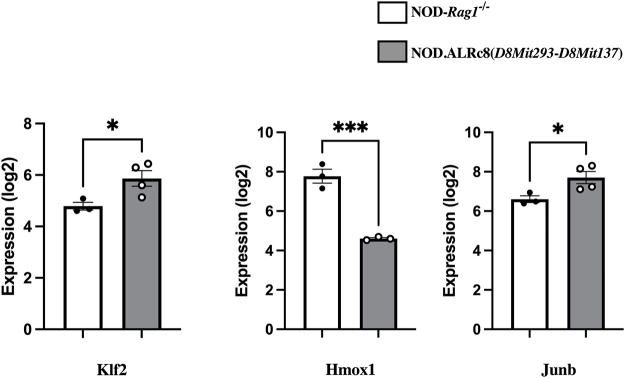
Transcriptome analysis identified genes differentially expressed in islets from insulitis-free NOD-*Rag1* ^*−/−*^ and NOD. ALRc8 (*D8Mit293*-*D8Mit137*) mice. Shown here are the expression of *Klf2*, *Hmox1* and *Junb* in islets from NOD-*Rag1* ^−/−^ (open bars, *n* = 3 pools) and NOD. ALRc8 (*D8Mit293*-*D8Mit137*) (Grey bars, *n* = 4 pools), respectively compared using an unpaired *t* test. *p < 0.05. ***p < 0.001.

Transcriptome analysis revealed over 3,000 transcripts were differentially expressed in the islets of these two mouse strains (threshold values of |log2(FC)|>0.5, *p*-value <0.05), of which 164 were located on Chr 8. Figure 8 shows some of these genes that are of interest (discussed below). The NOD. ALRc8 (*D8Mit293*-*D8Mit137*) and NOD-*Rag1* ^*−/−*^ islet gene expression array data are available at https://www.ncbi.nlm.nih.gov/geo/(Accession number: GSE206705).

## Discussion

AL is a toxic glucose analogue that is transported into and accumulates in rodent pancreatic β-cells by glucose transporter 2 (GLUT2). The β-cytotoxic action of this compound is mediated by ROS. Once inside β-cells, AL forms a conjugate with glutathione and is then reduced by thiol reductases (e.g., glutaredoxins) to form dialuric acid (Washburn and Wells, 1997). Autoxidation of dialuric acid generates superoxide radicals, hydrogen peroxide and, in a final iron-catalyzed reaction step, hydroxyl radicals. Hydroxyl radicals are ultimately responsible for death of the β-cells and the ensuing state of insulin-requiring, ALD. Due to the particularly low antioxidative defense capacity of rodent β-cells and the proposed role of ROS in the development of both Type 1 and Type 2 diabetes, ALD has been a useful model to understand mechanisms of β-cell failure. Indeed, the ALR mouse strain that was developed to resist ALD is also resistant to induction of T2D (Sekiguchi et al., 1991; Mathews et al., 2004), as well as spontaneous autoimmune diabetes (Mathews et al., 2003; Mathews et al., 2005; Chen et al., 2011a; Chen et al., 2011b). Earlier genetic analyses for ALR-derived resistance to T1D identified *mt-Nd2* of the mtDNA as well as loci on Chr 17 (*Idd16*), Chr 8 (*Idd22*), and Chr 3 (*Susp*) (Mathews et al., 2003; Mathews et al., 2005; Pomerleau et al., 2005).

Our previous study utilized a cohort of 296 reciprocal F2 hybrids from outcrosses of ALR and NOD mice. This population was used to identify the unique genetic resistance of the ALR strain to ALD. Four loci were linked to ALD, including three in the nuclear genome (Chr 2, 3, and 8) and one in the mtDNA (*mt-Nd2*). The ALR genome provided diabetes resistance at both *mt-Nd2* and a defined region on Chr 8 that has an overlapping confidence interval with *Idd22* (Chen et al., 2008b). In the current study with a larger F2 cohort (*n* = 678) we confirmed both of these linkages as well as a Y-linked trait. However, the results here with a larger cohort of F2 mice failed to replicate the independent linkage on Chr 2 or the linkage to Chr 3 while providing suggestive evidence for ALR-derived protective loci on Chr 4, 6, 7, 8, and 13. Interestingly, one protective linkage on Chr 5 of the NOD genome was identified. Further, both intragenomic and intergenomic epistasis were uncovered. The intergenomic epistasis for ALD resistance between ALR alleles at *mt-Nd2* and Chr 8 confirmed our previous observation using a congenic and conplastic mouse approach that NOD. mt^ALR^-*Idd22* mice were more resistant to ALD than either NOD. mt^ALR^ or NOD. *Idd22* (Chen et al., 2008a).

The unique SNP and resulting allele/allotype in the mtDNA of ALR, *mt-Nd2* ^*a*^, was previously associated with resistance against ALD (Chen et al., 2008b) and confirmed in the current study (Table 2). In contrast, the NOD *mt-Nd2* ^*c*^ allele has been associated with elevated mt ROS production (Gusdon et al., 2007; Gusdon et al., 2008) and β-cell sensitivity to free radical mediated (hydrogen peroxide and alloxan) as well as autoimmune destruction (Chen et al., 2008a; Chen et al., 2011b). The resistance contributed by *mt-Nd2* ^*a*^ to the potent free radicals generated by AL may be the result of suppressed endogenous mt free radical production (Mathews et al., 2005; Gusdon et al., 2007; Gusdon et al., 2008) and/or a higher level of antioxidant defenses unique to ALR pancreatic islets (Mathews et al., 2001; Mathews et al., 2005). Indeed, *mt-Nd2* ^*a*^ was also mapped to be protective against spontaneous T1D in mouse models (Mathews et al., 2005), and a corresponding human *mt-ND2* ^*a*^ (mtDNA D haplotype) was associated with resistance to T1D in humans (Uchigata et al., 2002) as well as to T2D in certain human populations (Liou et al., 2012). Therefore, the mechanisms underlying *mt-Nd2* ^*a*^ *-*associated β-cell resistance are a possible new target to preserve β-cell survival in T1D and potentially in T2D.

The Chr 8 ALD-resistance locus was mapped previously using a smaller F2 population of 296 mice to have an ALR-derived AL-resistance locus (Chen et al., 2008b). In a separate study using (NODxALR)F1 x NOD backcross 1 mice, the ALR allele on Chr 8, denoted as *Idd22,* was mapped to be protective against spontaneous T1D (Mathews et al., 2003) and later confirmed using congenic analysis (Whitener et al., 2017). These two Chr 8 linkages are overlapping (Chen et al., 2008b). With the current larger F2 sample size, the ALD-resistant linkage on Chr 8 is further confirmed (Figure 1). We have previously created congenic mice to examine the increased threshold for AL when ALR alleles on Chr 8 were introgressed onto the NOD background (Chen et al., 2008b). In the current study, we further shortened the congenic interval to a proximal region between 36.2 Mb and 77.8 Mb. The proximal short congenic mice show the same elevated threshold for AL resistance as the previous longer congenic (Figure 3), suggesting the gene or genes that contribute to resistance are in this proximal region. Consistent with this result, the peak of Chi-squares on Chr 8 is located within this region at 77 Mb (Figure 5), with 95% confidence interval between markers *D8Mit205* (51.9 Mb) and *D8Mit252* (93.48 Mb). Previously we determined, using congenic mice, that the mechanism of *Idd22*-mediated autoimmune diabetes resistance is through inhibition of autoreactive T cell extravasation through the vascular endothelium (Whitener et al., 2017). This was performed in NOD with the ALR allele of *Idd22* and the NOD allele of *mt-Nd2* excluding the possibility of observing the important intergenomic interaction between *Idd22* and *mt-Nd2* that provides protection to beta cells [Figure 5 and (Chen et al., 2008b)]. The fact that ALR and NOD mtDNA differ only at the *mt-Nd2* SNP allowed us to analyze the interaction of *mt-Nd2* with ALD-linked nuclear loci by stratification of the 678 F2 cohort by *mt-Nd2* genotype. The Chr 8 ALD-linkage was mapped only in the group of mice with *mt-Nd2* ^*a*^, ergo, the effect of the allele inherited from ALR (R) on Chr 8 requires the co-existence of *mt-Nd2* ^*a*^ to provide protection against ALD (Figure 5). Conplastic and congenic NOD. *mt* ^*ALR*^ *-Idd22* mice exhibited enhanced resistance to ALD, further supporting the intergenomic epistasis (Chen et al., 2008a).

Disease endotypes have been attributed to interactions between mitochondria and the nucleus. Proper mitochondrial function necessitates expression of genes from the nuclear and mitochondrial genomes. An important factor coordinating these two compartments is retrogenic signals that radiate from the mitochondria in response to metabolism or stress to impact gene expression and influence pathology (Cannino et al., 2007; Kim et al., 2018; Walker and Moraes, 2022). Genetic cooperation between the nucleus and mitochondria has been demonstrated to impact diabetes related phenotypes such as glucose metabolism and insulin sensitivity using mouse models (Sammy et al., 2021). The data presented here demonstrate directly that intergenomic epistasis controls beta cell sensitivity to β-cell death and diabetes onset.

We have previously associated *mt-Nd2* ^*c*^ with increased mtROS production (Gusdon et al., 2007; Gusdon et al., 2008). The interaction between Chr 8 and *mt-Nd2* might localize to a gene or genes on Chr 8 that control the dissipation of ROS or mitochondrial function to suppress mtROS production. When endogenous ROS produced at mitochondria is suppressed with *mt-Nd2* ^*a*^, we propose that the free radical dissipation capacity of the ALR-allele on Chr 8 will be set free to extinguish the exogenous free radicals induced by AL. Since the ALD-linked Chr 8 locus overlaps with T1D-protective *Idd22* (Mathews et al., 2003), and *mt-Nd2* ^*a*^ was also associated with T1D (Mathews et al., 2005), the interaction between *mt-Nd2* and Chr 8 appears to regulate β-cell survival during development of diabetes. We have compared gene expression of islets from NOD. *Rag1* ^*−/−*^ and NOD.ALRc8 (*D8Mit293*-*D8Mit137*) mice using transcriptome arrays. Among 164 differentially expressed Chr 8 transcripts between these two strains there are some candidates linked to mitochondrial function, ROS levels, or cell death. A few examples (Figure 8): *Klf2* (73 Mb) encodes Kruppel-like factor 2, a transcription factor involved in endothelium function, T cell development and immune activation and is closely related with mitochondrial function (Coon et al., 2022). *Hmox1* (75.1 Mb) encodes Heme Oxygenase 1, a known antioxidative enzyme exhibited higher expression in NOD islets compared to NOD. ALRc8(*D8Mit293*-*D8Mit137*). Induced expression of Hmox1 protects NOD mice from T1D and islets form destruction (Pileggi et al., 2001; Pogu et al., 2019). However, the increased expression is likely an indicator of increased mitochondrial oxidative stress in NOD compared to the *Idd22* congenic strain (Johnson and DeMars, 2004) as opposed to *Hmox1* being an ideal candidate gene. *Junb* (84.98 Mb) encodes a subunit of AP-1 transcription factor. JunB has been shown to be protective against mitochondrial stress in human lymphoma cells (Son et al., 2010), and structurally, JunB can localize in mitochondria (Liu et al., 2018). These possible candidate genes locate close to the peak linkage (77 Mb) on Chr 8 (Figure 5) and are within the 95% confidence interval. These may contribute to ALD resistance by impacting the interactions between Chr 8 and *mt-Nd2*. Further studies are needed to develop β-cell lines with both *mt-Nd2* ^*a*^ and Chr 8 (ALD-linkage/*Idd22*) to isolate the effect of this interaction at β-cell level.

The mtDNA haplotype also affects interactions of Chr 8 with Chr 2 (Figure 6). Although the linkage on Chr 2 was not alone significant as an independent linkage in the larger cohort studied here, a strong epistatic interaction between Chr 2 and Chr 8 was detected in the full population of F2 mice (Figures 4, 6; Table 3). The interaction between Chr 2 and Chr 8 appeared using multiple regression modeling [drop one QTL at a time ANOVA analysis (Figure 4; Table 3)]. This epistatic interaction is affected by the *mt-Nd2* allele (Figure 6). Dominant protection of ALR genome on Chr 8 suppresses the ALD sensitivity linked to the heterozygous genotype on Chr 2. Therefore, the negative heterosis conferred by the linkage on Chr 2 is only observed when Chr 8 is homozygous for the NOD genotype resulting in heightened ALD incidence, and is at the highest when *mt-Nd2* is also NOD type. As the Chr 2 linkage alone is not significant, it is likely that the ALR allele on Chr 8 provides too powerful a resistance and outplays sensitivity conferred by the Chr 2 linkage. The peak on Chr 2 is denoted by marker *rs3681744* at 121 Mb. An interesting candidate gene near *rs3681744* is *28S ribosomal protein S5, mitochondrial* (*Mrps5*). Expression analysis demonstrates that *Mrps5* transcripts are at a significantly lower level in isolated NOD. *Rag1* ^*−/−*^ islets compared to NOD.ALRc8(*D8Mit293-D8Mit137*) islets. As this regulates mitochondrial protein it may affect the Chr2-Chr8-*mt-Nd2* interaction to prevent AL-induced mitochondrial damage through control of translation of genes encoded on the mitochondrial genome, including *mt-Nd2* (Titova et al., 2020).

The NOD linkage (D) on Chr 6 contributes dominant AL sensitivity whereas the NOD contribution on Chr 8 is recessive (Figure 2). A Chr 8-Chr 6 was not detected in the full population of 678 F2 mice not stratified by mt-ND2 genotype (Figure 7A). The NOD allele (D) on Chr 6 contributes dominant AL sensitivity but the NOD susceptibility contribution on Chr 8 appears to act recessively (Figure 2). However, a distinct interaction was revealed when the population of F2 mice was stratified by mt genotype. In segregants expressing the NOD *mt-Nd2* ^*c*^ *,* homozygous D type on both Chr 8 and 6 synergistically contributed to ALD susceptibility (Figure 7B). On the contrary, in segregants expressing the ALR *mt-Nd2* ^*a*^, homozygosity for the NOD Chr 8 linkage (DD) exhibited maximum susceptibility in combination with homozygosity for the ALR linkage (RR) on Chr 6 (Figure 7C). The peak linkage on Chr 6 is at marker *D6Mit184* (53.2 Mb), with 95% confidence interval between markers D6Mit48 (31.9 Mb) and D6Mit323 (86.85 Mb). Analysis of differentially expressed genes in the islets from NOD. *Rag1* ^*−/−*^ compared to NOD. ALRc8 (*D8Mit293-D8Mit137*) identified *Hdac11* (histone deacetylase 11) at 91 Mb, as expressed at lower level in the islets of ALD-susceptible NOD. *Rag1* ^*−/−*^ mice. *Hdac11* has been reported to affect mitochondrial function (Hurtado et al., 2021). At 98.9 Mb, a gene *Foxp1*, encodes *forkhead box P1* is a transcription factor for mtDNA binding protein (Wang et al., 2019) and has reduced expression in ALD-resistant NOD.ALRc8(*D8Mit293-D8Mit137*) islets. As AL can induce oxidative stress-mediated mtDNA damage (Driggers et al., 1997), a reduction in *Foxp1* may protect against β-cell death by reducing replication of damaged mtDNA allowing for repair to occur. Although these genes are outside of the 95% confidence interval on Chr 6, given their association with mitochondria, these remain interesting candidates.

In autoimmune T1D genes may impact both the immune system and non-immune cells (i.e., β-cells) (Barrett et al., 2009; Inshaw et al., 2020). These results therefore do not exclude the possibility that *Idd22* affects both β-cells and immune cells either by different genes in this region or by a single common gene. In fact, *Idd22* alone confers resistance to spontaneous T1D in NOD (Mathews et al., 2005) by hampering autoreactive T cell trafficking to islets (Whitener et al., 2017), while islet susceptibility to *in vitro* autoimmune attack is not affected (Whitener et al., 2017). This is another example of T1D as a complex and multifactorial disease (Driver et al., 2012; Chen Y. G. et al., 2018).

With the emerging data from genetic studies since tools like GWAS became available, genes and loci involved in T1D and T2D have been identified. Genes identified by GWAS to be involved in diabetes have been linked with mitochondria or mitochondrial function as well as β-cell death. An example is GLIS Family Zinc Finger 3 (*GLIS3*), which is associated with both T1D and T2D (Wen and Yang, 2017). Knockdown of *GLIS3* induced BAX translocation to mitochondria, increasing cleaved caspase 3 and 9, and cytochrome c release, therefore potentiates cytokine-induced β-cell death *via* the mitochondrial pathway of apoptosis (Nogueira et al., 2013). *PDX1*, another gene associated with both T2D (Hani et al., 1999) and monogenic diabetes of the young (MODY) (Stoffers et al., 1997), is thought to control β-cell function by regulation of mitophagy, a key mechanism for mitochondrial turnover and quality control (Ding and Yin, 2012), through controlling of the T1D-associated gene *CLEC16A* (Soleimanpour et al., 2014; Soleimanpour et al., 2015). The *mt-ND2* SNP *rs28357984* (5178 A/C) in the human mitochondrial genome that corresponds to the mouse *mt-Nd2*, was also associated with both human T1D (Uchigata et al., 2002) and T2D (Liou et al., 2012). The intergenomic epistasis was studied in mt-exchange mouse model of fatty liver disease (Betancourt et al., 2014). In human T1D, mt-nuclear epistasis is associated with patients’ BMI (Ludwig-Slomczynska et al., 2020). Interestingly, in this study, the mtDNA SNP that interacts with nuclear genes was also in *mt-ND2* (Ludwig-Slomczynska et al., 2020). Both fatty liver disease and BMI are factors associated with T2D. Therefore, mitochondria might be a common factor in the pathogenesis of both T1D and T2D through affecting β-cell fitness. Since both types of diabetes are multifactorial diseases, mt-nuclear interactions may play pathogenic or protective roles in both diseases. To the best of our knowledge, the current study is the first to investigate the role of mt-nuclear interactions in the control of β-cell sensitivity to ROS and development of diabetes.

In summary, using a F2 cohort generated by outcrosses of the NOD and ALR mouse strains, we detected loci that affect pancreatic β-cell sensitivity to free radical-generating chemical alloxan on Chromosomes 4, 5, 6, 7, 8, and 13, as well as *mt-Nd2* of the mtDNA. Further, we identified that both intra- and intergenomic epistasis contribute to resistance or sensitivity of β-cell death in this model. This is the first report to demonstrate that interactions of the mitochondrial genome and the nuclear genome can regulate diabetes. Mitochondria are pivotal for β-cell function and insults or dysfunction of this organelle may impact pathogenesis (Uchigata et al., 2002; Chen et al., 2011b; Liou et al., 2012; Chen et al., 2017; Chen J. et al., 2018). Therefore, epigenetic interactions that affect β-cell resistance to free radical stress may alter the development of both T1D and T2D.

## Data Availability

The datasets presented in this study can be found in online repositories. The names of the repository/repositories and accession number(s) can be found below: https://www.ncbi.nlm.nih.gov/geo/, GSE206705.
